# Combined Kidney-Liver, Heart-Liver, and Kidney-Pancreas Transplantations from a Single Deceased Donor

**DOI:** 10.1155/2012/849619

**Published:** 2012-10-11

**Authors:** Matteo Ravaioli, Matteo Serenari, Matteo Cescon, Sofia Martin Suarez, Alessandro Cucchetti, Giorgio Ercolani, Massimo Del Gaudio, Fausto Catena, Cristina Morelli, Giorgio Arpesella, Antonio Daniele Pinna

**Affiliations:** ^1^Department of Liver and Multi-organ Transplantation, Sant 'Orsola-Malpighi Hospital, University of Bologna, Via Massarenti 9, 40138 Bologna, Italy; ^2^Department of General Surgery and Transplantation, Sant 'Orsola-Malpighi Hospital, University of Bologna, Via Massarenti 9, 40138 Bologna, Italy; ^3^Heart and Lung Transplantation Program, Department of Cardiac Surgery, Sant 'Orsola-Malpighi Hospital, University of Bologna, Via Massarenti 9, 40138 Bologna, Italy

## Abstract

Splitting the liver for two adults to increase the donor pool is still a debated issue, especially for combined organ transplantation. We described a case of liver-splitting procedure for two adults, which was successful even in the presence of combined organ transplantation. Three adult combined organ transplantations from one deceased donor were performed, with, use of split liver grafts in two patients: a combined heart-right split liver, a left kidney-left split liver, and a right kidney-pancreas transplantation. Despite a not perfect match between the graft type and recipient, the prevention of small-for-size syndrome by ligature of the splenic artery, and/or hemiportocaval shunt in the patient receiving the left split liver, and the maximal reduction of ischemia time were the main factors contributing to the success of the procedure. This is the first report of combined heart and split liver in two adults which may suggest new strategies for organ transplantations.

## 1. Case Report

Split liver transplantations for two adults are a demanding procedure, and an allocation system, which grants priority to sickest patients, may discourage it [1–3]. The surgical and medical complications related to combined organs transplantation are additional reasons to avoid split liver for such procedures. On the other hand, waiting list mortality due to the shortage of deceased donors spurs strategy to make more organs available for transplantation, and split liver transplantation for two adults even with combined organs may be a safe possibility [4].

We agree, according to our personal experience, to split for two adults, dividing the left lateral segment (segments II-III, LLS) from segments IV to VIII and the caudate lobe, preserving the right graft for the sickest recipient on waiting list and with more demanding procedure, like combined heart-liver transplantation. The left lateral segment should be transplanted in a selected recipient, and a combined kidney-liver transplantation could not be a limitation.

**Figure 1:**
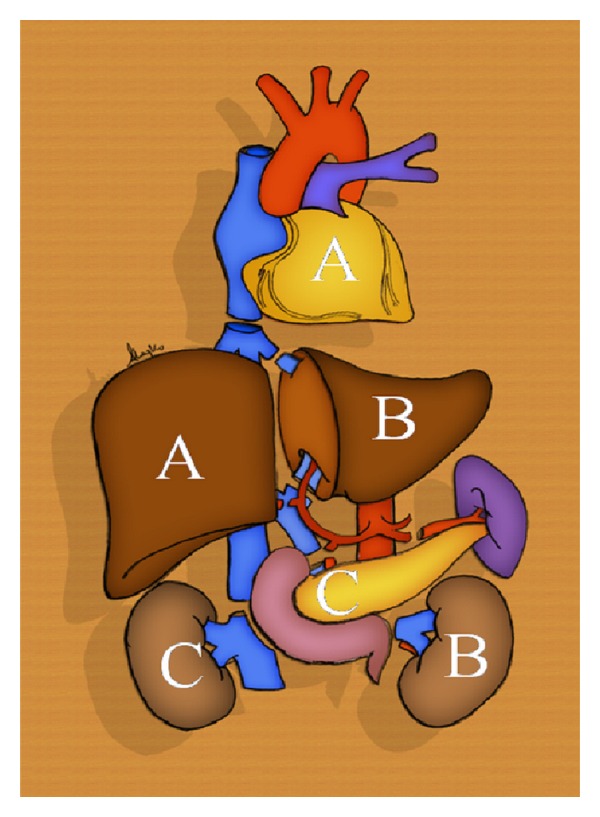
Organ harvesting from the same deceased donor to permit three combined organ transplantations: heart-right liver (A); left kidney-left liver (B); pancreas-right kidney (C).

Our center performed a successful combined heart-liver and kidney-liver transplantation from the same donor, whose liver was divided in situ (Figure 1).

The deceased donor was 38-year-old male, 95 kg × 180 cm, who died of a head trauma. After the cardiothoracic surgeons, who first started with preparation of the heart and great vessels, the liver surgeon performed a liver biopsy to exclude any steatosis and the split procedure was performed in situ with a complete division of the liver parenchyma, as previously described. Another team prepared the pancreas, which later was successfully transplanted combined with the right kidney at another center. 

The right graft (1450 mg) was utilized for a recipient (75 kg × 175 cm), who suffered from familial amyloid polyneuropathy (FAP) with a cardiac insufficiency and autonomic dysfunction.

The heart transplantation was performed with the standard technique [5], and cardiopulmonary bypass (CPB) was maintained for nearly 45 minutes until optimal cardiac and hemodynamic performances were obtained. Afterwards, patients were weaned from CPB, hemostasis was performed, and temporary pacemaker leads and drains were positioned. The chest was left open, just covered with a gauze during the whole abdominal procedure.

The liver transplantation was performed preserving the vena cava with the “piggy-back” technique and without any bypass. A T tube was used for the biliary anastomosis to reduce the risk of biliary fistula, and the cold ischemia time was six hours.

The LLS was utilized in a female with polycystic liver and kidney disease (55 kg × 168 cm). To reduce the risk of small-for-size syndrome (LLS = 350 mg; GRWR = 0.64) a catheter was placed inside the portal vein and the portal venous pressure was measured. The portocaval gradient after LLS reperfusion was 26 mmHg. It decreased to 18 mmHg after the ligation of the splenic artery. Such as a pressure was considered too high, and thus we proceeded with a hemi-portocaval shunt, leading to a final portal pressure of 10 mmHg [6].

The T tube was not applied for the duct-to-duct biliary anastomosis, due to the low size of the biliary duct of the graft and recipient. The ischemia time was seven hours. At the end of the liver transplantation, the left kidney was transplanted in the right iliac fossa, using the external iliac vessels.

After laparotomy closure, the chest was closed in the conventional fashion. 

**Figure 2:**
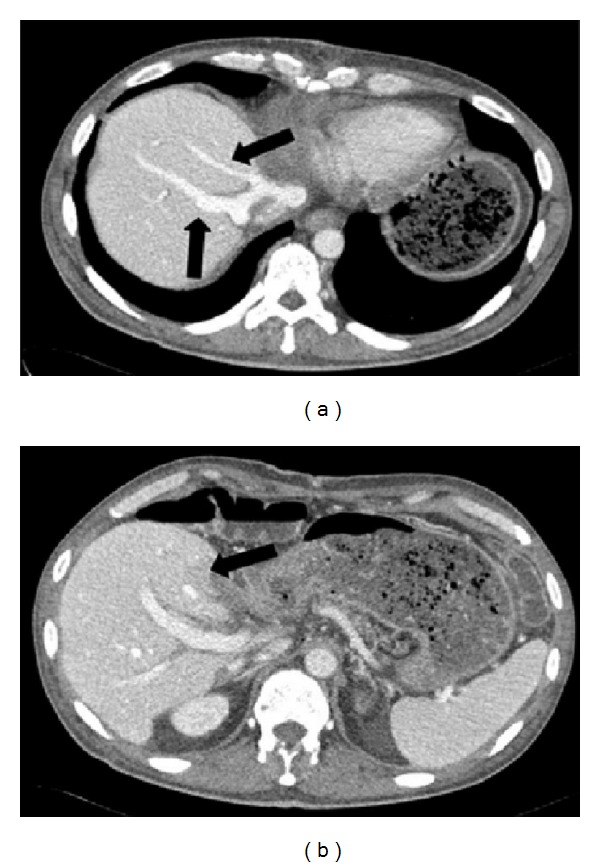
CT scan of combined heart-right liver transplantation: arrows of Figure 2(a) show middle and right hepatic veins; arrow of Figure 2(b) shows the segment IV of the liver with normal perfusion.

The postoperative course was uneventful for the heart-right liver split transplantation, who was discharged after 20 days, and a CT-scan showed a normal perfusion of the liver, even of segment IV (Figures 2(a) and 2(b)).

**Figure 3:**
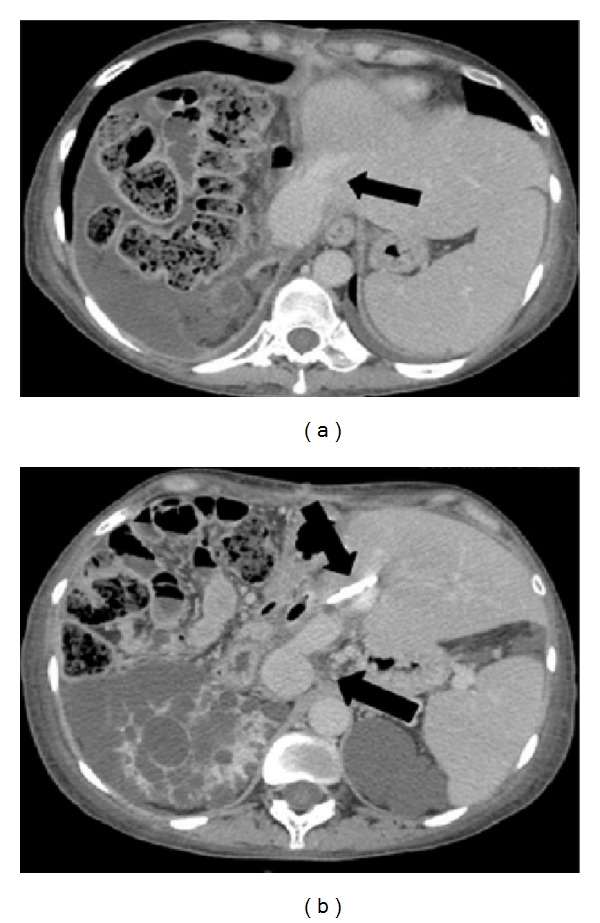
CT scan of combined left kidney-left liver transplantation: arrow of Figure 3(a) shows the left hepatic vein; arrows of Figure 3(b) show the biliary stent and the portocaval shunt.

The recipient of combined kidney-left liver transplantation experienced a biliary leak from the cut surface, which needed a reoperation on day 2 to suture the fistula; furthermore, the patient had a stenosis of the biliary anastomosis, which needed the placement of a plastic stent by endoscopy. The patient did not experience any small-for-size syndrome; the blood bilirubin level was always normal, ascites disappeared after two weeks, and she did not develop encephalopathy. She was discharged after four weeks and the CT scan showed a patency of the portocaval shunt (Figures 3(a) and 3(b)).

At the best of our knowledge, this is the first report of combined heart-liver and kidney-liver transplantation for two adults using the same donor and applying the split procedure.

Split liver transplantation for an adult, and a child achieves results equivalent to whole liver transplantation, while for two adults it is still not an established procedure for two principal problems: small-for-size syndrome and biliary complications. Furthermore, combined organ transplantation, heart-liver or kidney-liver, may increase the risk of surgical complications.

We considered the possibility of splitting for two adults, dividing the left lateral segments (LLS segments II and III) from segments IV to VIII and the caudate lobe [2, 3]. This type of procedure may be applied without accurate preoperative imaging studies, which are not available in peripheral hospitals. The right graft with the vena cava, the right branch of the hepatic artery, and the portal vein can be utilized for the sickest patient on list or for the patient with the more demanding procedure, like combine,s heart-liver transplantation. The left graft with the left hepatic vein, the left branch of the portal vein, and the left branch of the hepatic artery, along with the common hepatic artery and celiac axis could be allocated to an adult with no severe portal hypertension like our liver-kidney polycystic recipient, listed also for kidney transplantation. 

We believe the following to be important for the success of this procedure: application of the T tube in both grafts, if possible, for better early diagnosis and treatment of biliary complications; preventing the small-for-size syndrome with the ligature of the splenic artery and/or hemi-portocaval shunt in patient receiving LLS and reducing as much as possible the ischemic damage with low ischemia time. The decision about the portocaval shunt should be evaluated according to the portal vein pressure recorded after the graft reperfusion. The procedure is essential to reduce the congestion of the left graft, which may be wrongly confused with the liver regeneration on the CT scan and favor the increase of vascular resistance up to inducing arterial thrombosis and graft failure. There is not yet full consensus about the right time when closing the portocaval shunt.

Split liver transplantation may be possible and safe with combined transplantation if extended right graft is used for heart-liver transplantation and left graft used for selected recipient even in combined kidney-liver transplantation. The modulation of the portal flow is the key topic for the success of the left graft in combination of a low ischemia time. 

## Abbreviations

(SLT): Split liver transplantation
(LT): Liver transplantation
(LLS): Left lateral segment
(FAP): Familial amyloid polyneuropathy
(CPB): Cardiopulmonary bypass
(GW/RW): Graft weight/recipient weight
(CT-scan): Computer tomographyscan.
